# Supplementary material for: Flexible calorimetric flow sensor with unprecedented sensitivity and directional resolution for multiple flight parameter detection
Source: Nat Commun. 2024 Apr 10;15:3091. doi: 10.1038/s41467-024-47284-7 (PMC11006672; doi:10.1038/s41467-024-47284-7)
Supplement: Supplementary file 1 — Supplementary Information [file 41467_2024_47284_MOESM1_ESM.pdf]

Supplementary Information

Flexible Calorimetric Flow Sensor with Unprecedented Sensitivity and Directional Resolution for Multiple Flight Parameter Detection

Zheng Gong[1], Weicheng Di[2], Yonggang Jiang[1,3,*], Zihao Dong[1], Zhen Yang[1,4], Hong Ye[1], Hengrui Zhang[1], Haoji Liu[2], Zixing Wei[2], Zhan Tu[5], Daochun Li[2], Jinwu Xiang[2], Xilun Ding[1], Deyuan Zhang[1] & Huawei Chen[1]

[1]School of Mechanical Engineering and Automation, Beihang University, Beijing 100191, China

[2]School of Aeronautic Science and Engineering, Beihang University, Beijing 100191, China

[3]International Research Institute for Multidisciplinary Science, Beihang University, Beijing 100191, China

[4]Zhiyuan Research Institute, Hangzhou 310013, China

[5]Institute of Unmanned Systems, Beihang University, Beijing 100191, China

*Corresponding author. Email: Yonggang Jiang (jiangyg@buaa.edu.cn)

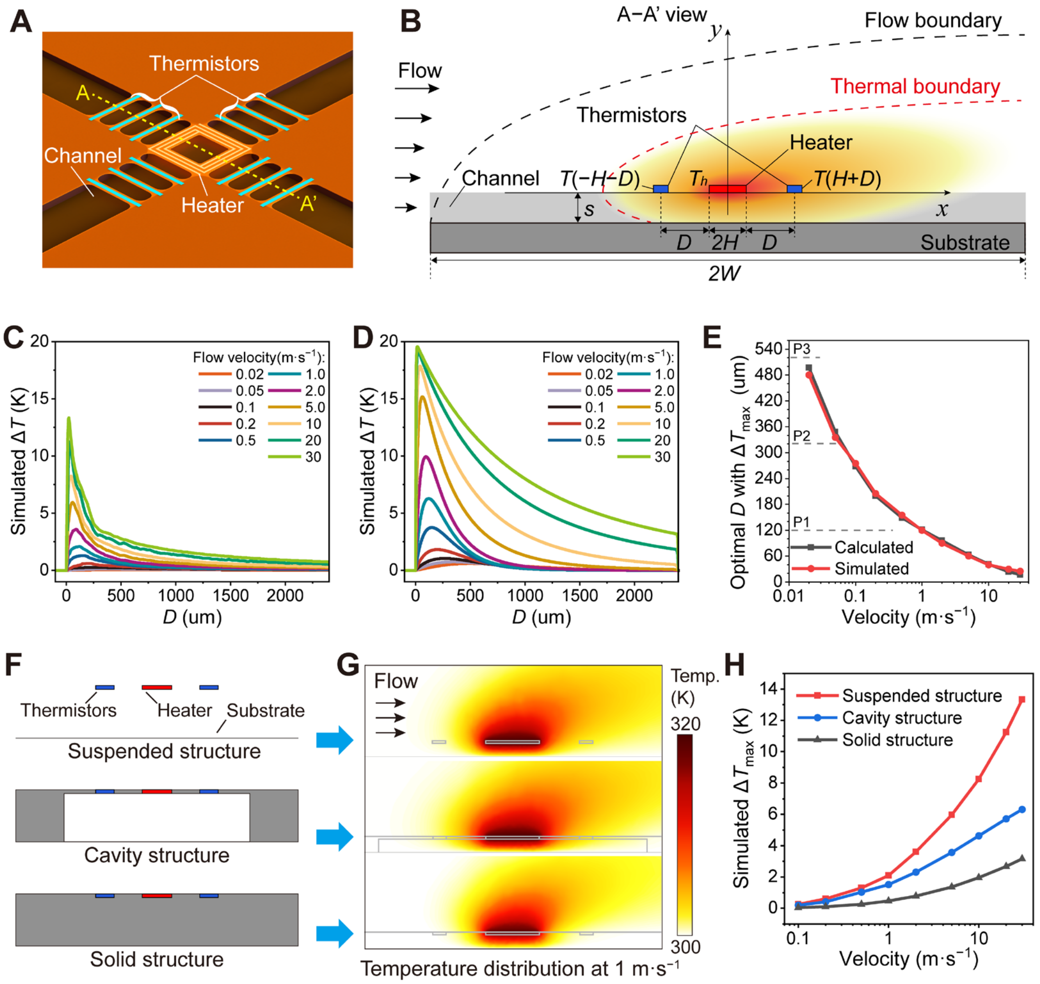

Supplementary Fig. 1. FCF sensing mechanism and modeling. (A) Schematic of the FCF sensor with the suspended structure. **(B)** A simplified one-dimensional model consists of a heater at the center of a channel with height *s* and overheated temperature T_h, and two symmetrically located upstream and downstream thermistors with temperature $T(-H-D)$ and $T(H+D)$, respectively. $2H$ is the Heater length and $2W$ is the channel length. The distance from the edge of thermistor to the center of heater is D. **(C)** Calculation and **(D)** simulation of the effect of distance D between the heater and thermistors on ΔT at different flow velocities (T_h = 20 K, $2W$ = 5 mm, $2H$ = 200 µm, s = 50 µm, D from 0 to 2.4 mm). **(E)** Simulation and calculation of the optimal distance D with maximum output ΔT_{max} at different flow velocities. **(F), (G)** Calorimetric flow sensors with three different structures of the same size and simulated local temperature distribution at 1 m·s^{-1}. **(H)** Comparison of the ΔT_{max} between three different structures with the suspended structure showing the highest sensitivity.

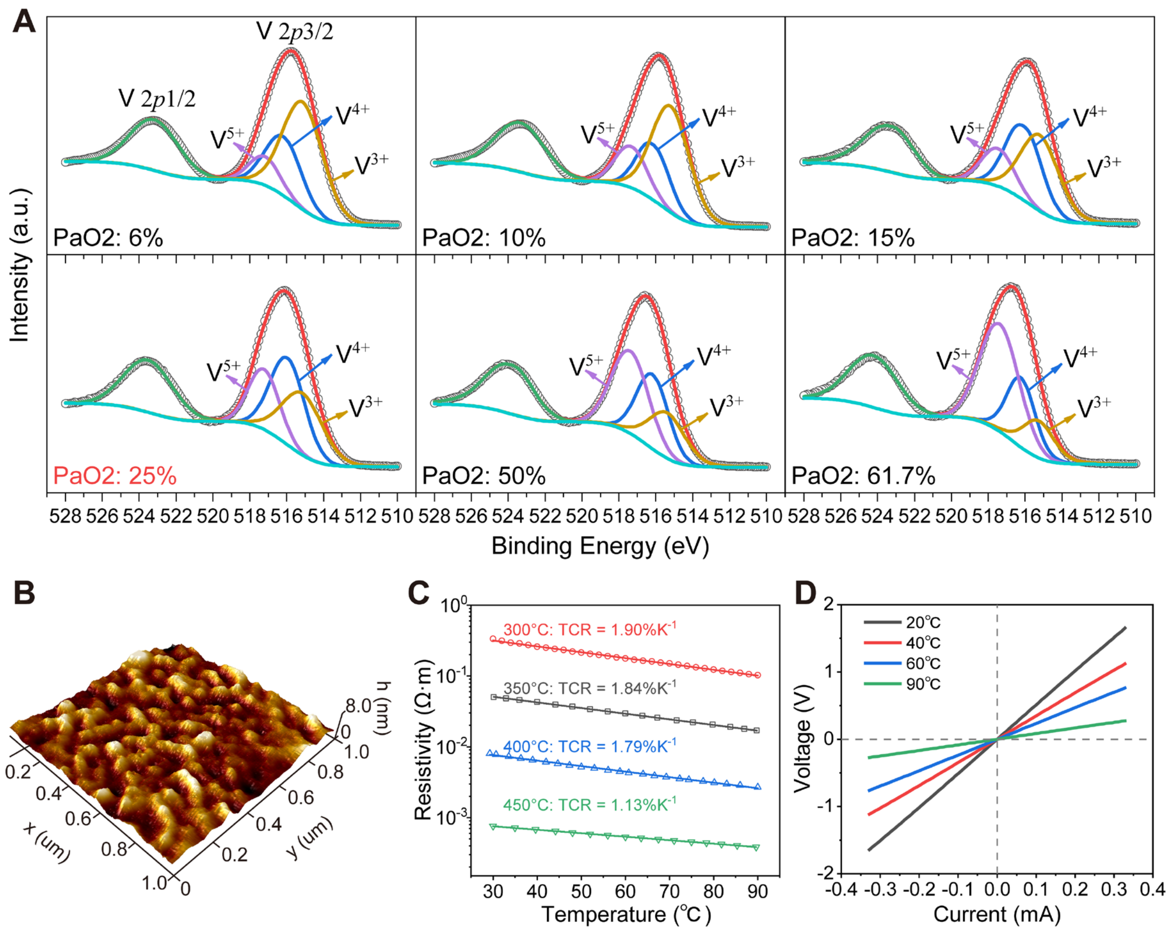

Supplementary Fig. 2. Preparation and characterisation of VO_x thin film. (A) XPS scans of V2*p* 3/2 of VO_x films after reactive ion beam sputtering process at different PaO2. As the PaO2 increases, the atomic ratio of V^{5+} increases and that of V^{3+} decreases. As more VO_2 (V^{4+}) means higher TCR, 25% PaO2 was used to maximize the component of VO_2. **(B)** AFM images of the surface morphology of VO_x film with the roughness *Ra* of 0.88 nm. **(C)** Temperature dependence of the resistance for the VO_x films at different annealing temperatures for 2 hours. The annealing temperature was chosen to be 300 °C to obtain a larger TCR. The resistivity of VO_x film is 0.33 Ω·m at 30 °C with a TCR of 1.9 $\%\cdot K^{-1}$. **(D)** V-I curves of the VO_x film over the temperature range of 20 to 90°C, exhibiting ohmic contact characteristics.

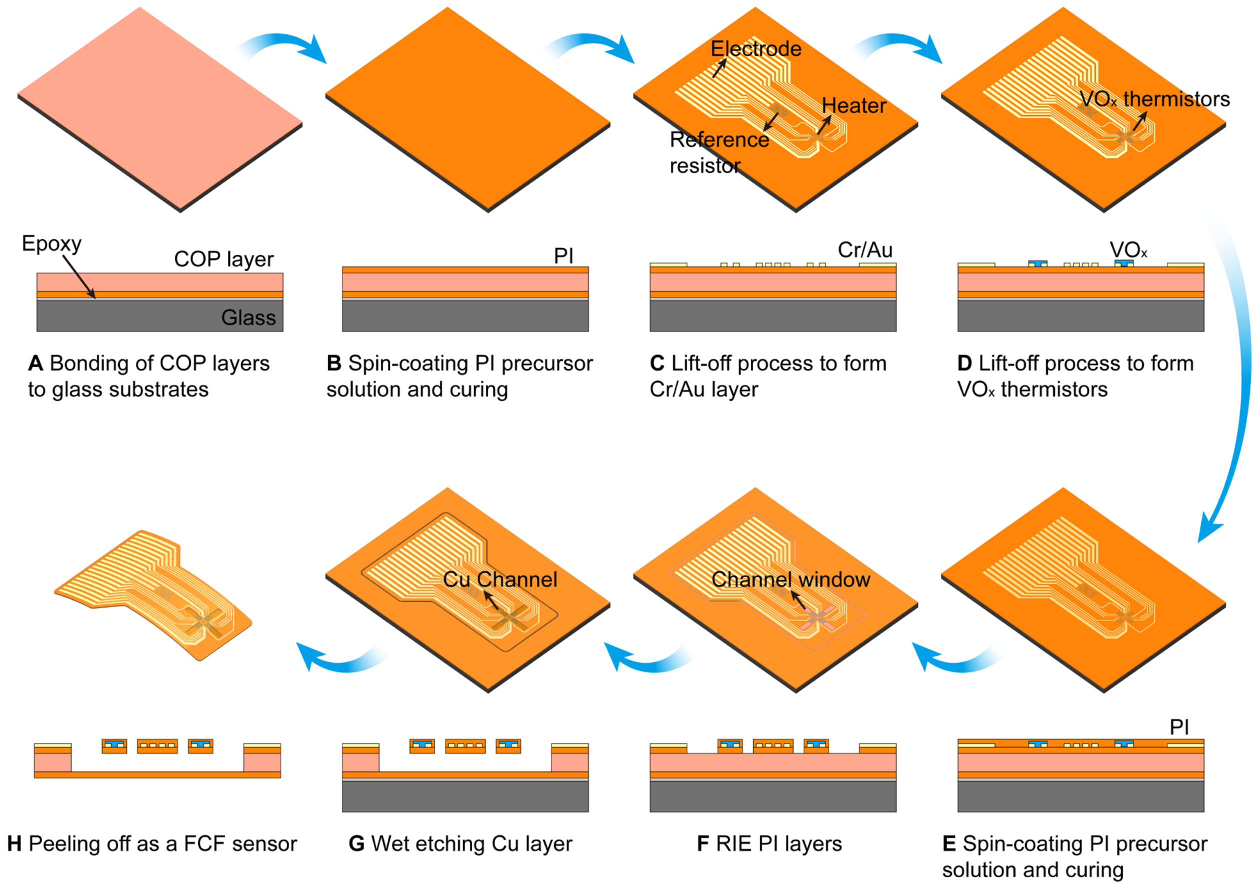

Supplementary Fig. 3. FCF sensor fabrication process. (A) Bonding of the COP layer to the glass substrate using high-temperature-resistant proxy. **(B)** Spin-coating and curing to form a PI supporting layer. **(C)** Lift-off process to form Cr/Au layer. **(D)** Lift-off process to form VO_x thermistors. **(E)** Spin-coating and curing to form a PI encapsulated layer. **(F)** RIE PI layers to expose electrodes and channel windows. **(G)** Wet etching exposed Cu of the COP layer to form the Cu channel. **(H)** Peeling off the monolithic FCF sensor from the glass substrate.

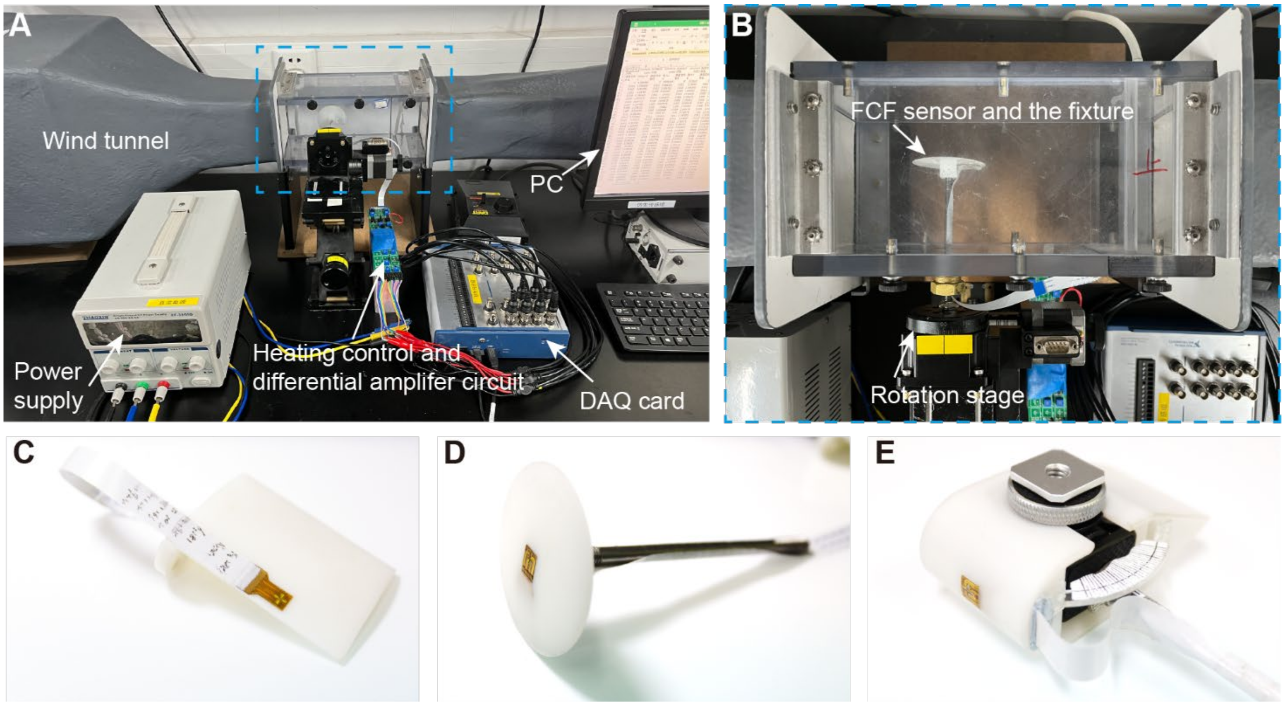

Supplementary Fig. 4. Experimental setup for evaluating the characteristics of the FCF sensor. (A) Components of the experimental setup. **(B)** Relative mounting position of FCF sensor in the wind tunnel. **(C)** FCF sensors mounted on the sharp plate for evaluating sensitivity, repeatability, and response time. **(D)** FCF sensor mounted in the middle of the slightly raised disk surface for directionality assessment. **(E)** FCF sensor mounted on the leading edge of the airfoil for AOA and AOS estimation.

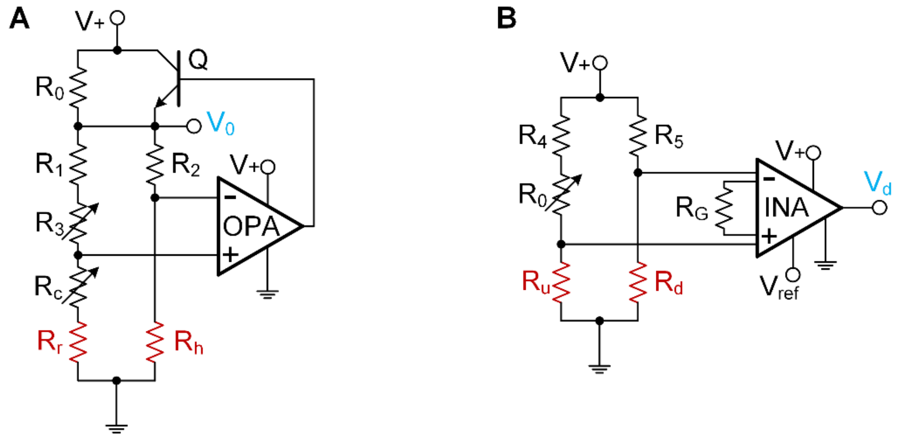

Supplementary Fig. 5. Schematic diagram of the calorimetry and heating circuit. (A) Constant temperature differential heating control circuit for FCF sensor with the on-chip reference ambient temperature sensor R_r, heater R_h, the external compensation resistor R_c, operational amplifier OPA, and NPN transistor Q. By adjusting the compensation resistor R_c, a constant temperature difference T_h = 20 K was maintained between the heater and the ambient temperature. V_0 represents the voltage loaded on the bridge. **(B)** Wheatstone bridge-based differential amplifier circuit with the on-chip VO_x thermistors upstream R_u, downstream R_d of the heater, and instrumentation amplifier INA. Where R_G denotes the gain resistor, V_+ denotes the supply voltage, V_{ref} denotes the reference voltage, and V_d denotes the differential voltage output.

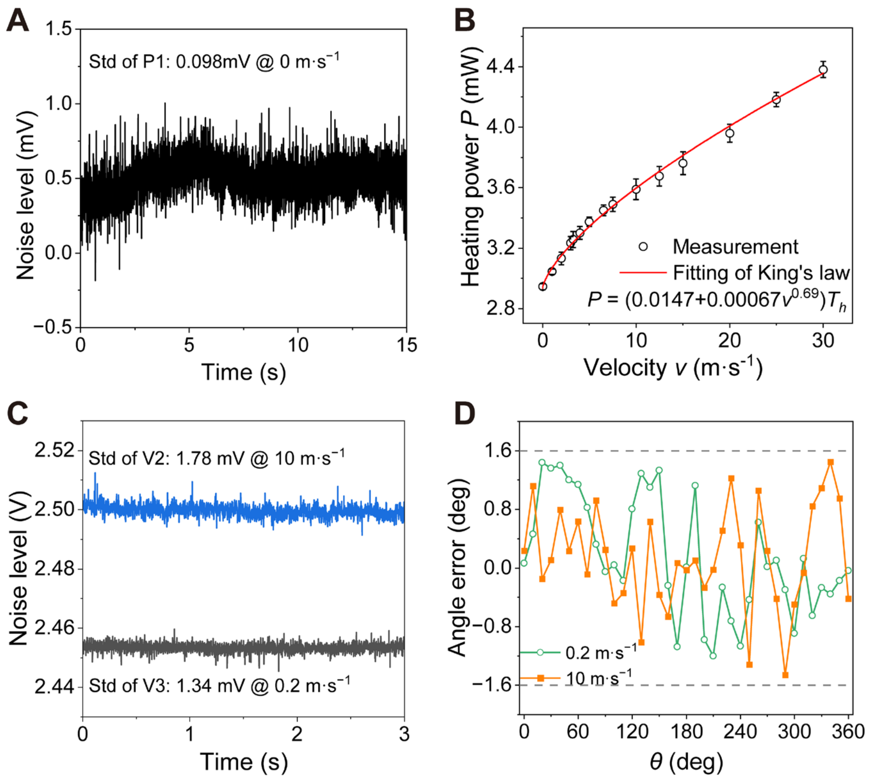

Supplementary Fig. 6. Noise levels, heater power, and angle errors of the FCF sensor. (A) Output voltage of P1 without wind with a standard deviation of 9.8×10^{-5} V as noise level. **(B)** Heating power in the flow velocity range of 0–30 m·s^{-1}. Error bars represent standard deviation, n = 3 independent replicates. **(C)** Noise levels of FCF sensor at 0.2 and 10 m·s^{-1} at an airflow angle of 180° airflow angle. **(D)** Angle errors of FCF sensors at 0.2 and 10 m·s^{-1}.

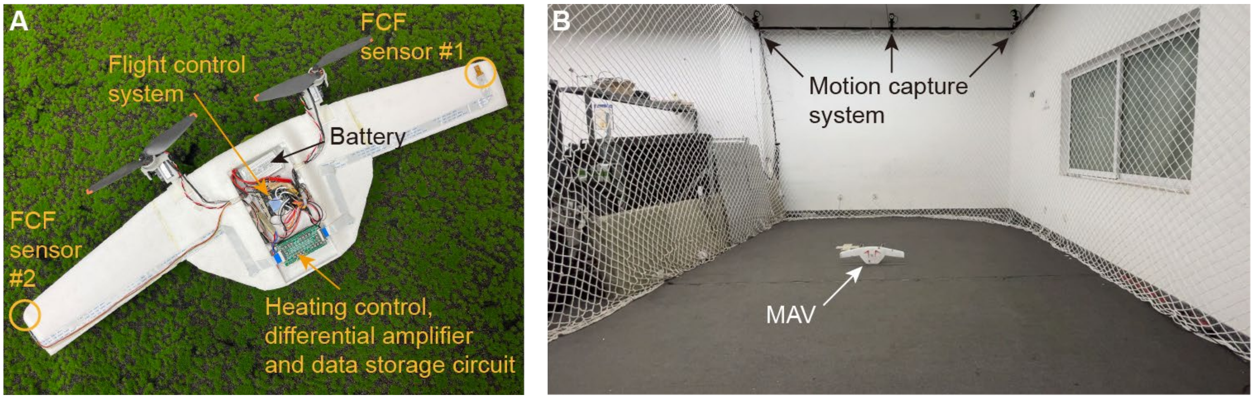

Supplementary Fig. 7. MAV installed FCF sensors and motion capture system. (A) Internal modules for the MAV, including the onboard electronics for control and sensing, and the power supply. **(B)** Indoor experimental field equipped with a motion capture system.

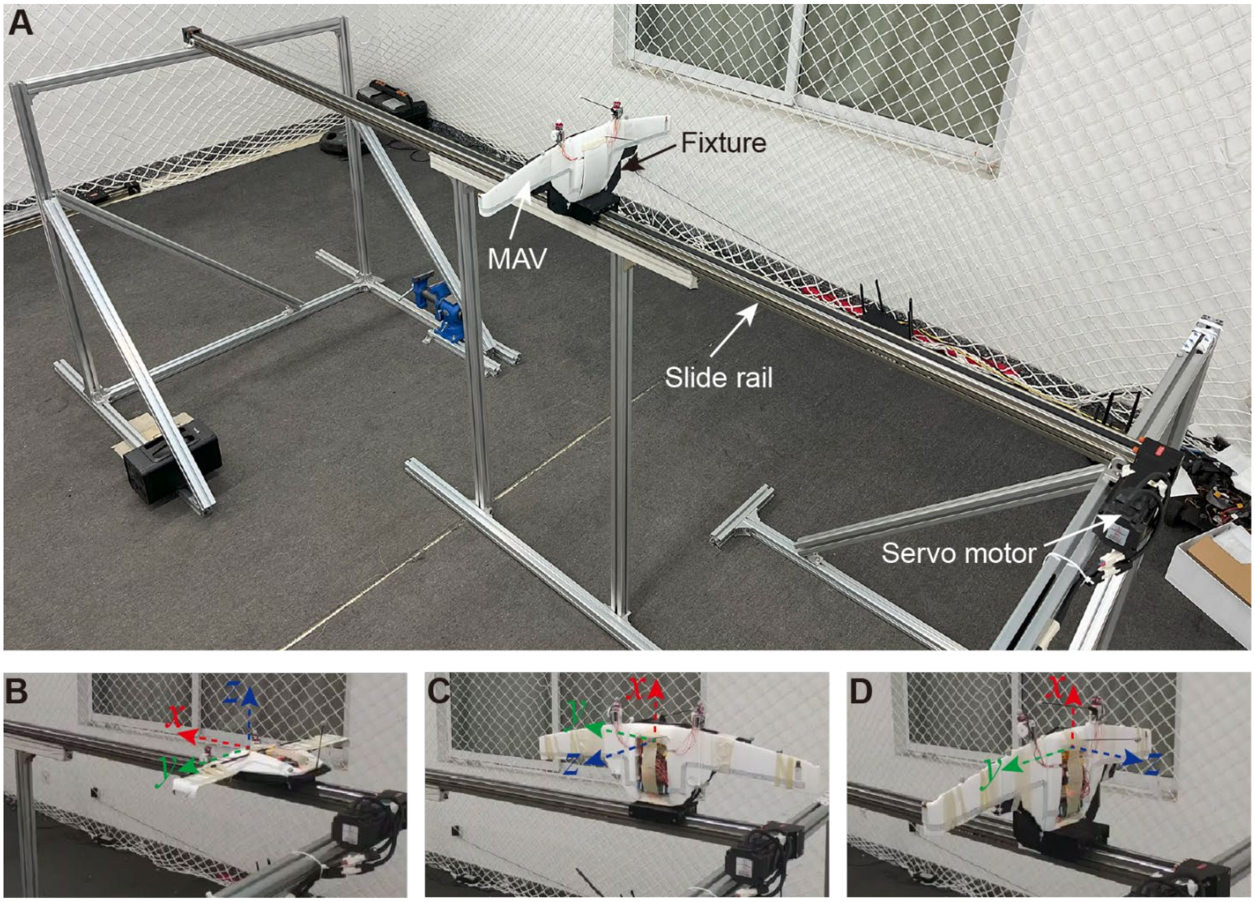

Supplementary Fig. 8. Experimental setup for MAV tri-axis motion. **(A)** Servo actuated slide system with MAV mounted. **(B)** MAV moving along the x-axis of the body coordinate system. **(C)** MAV moving along the y-axis of the body coordinate system. **(D)** MAV moving along the z-axis of the body coordinate system.

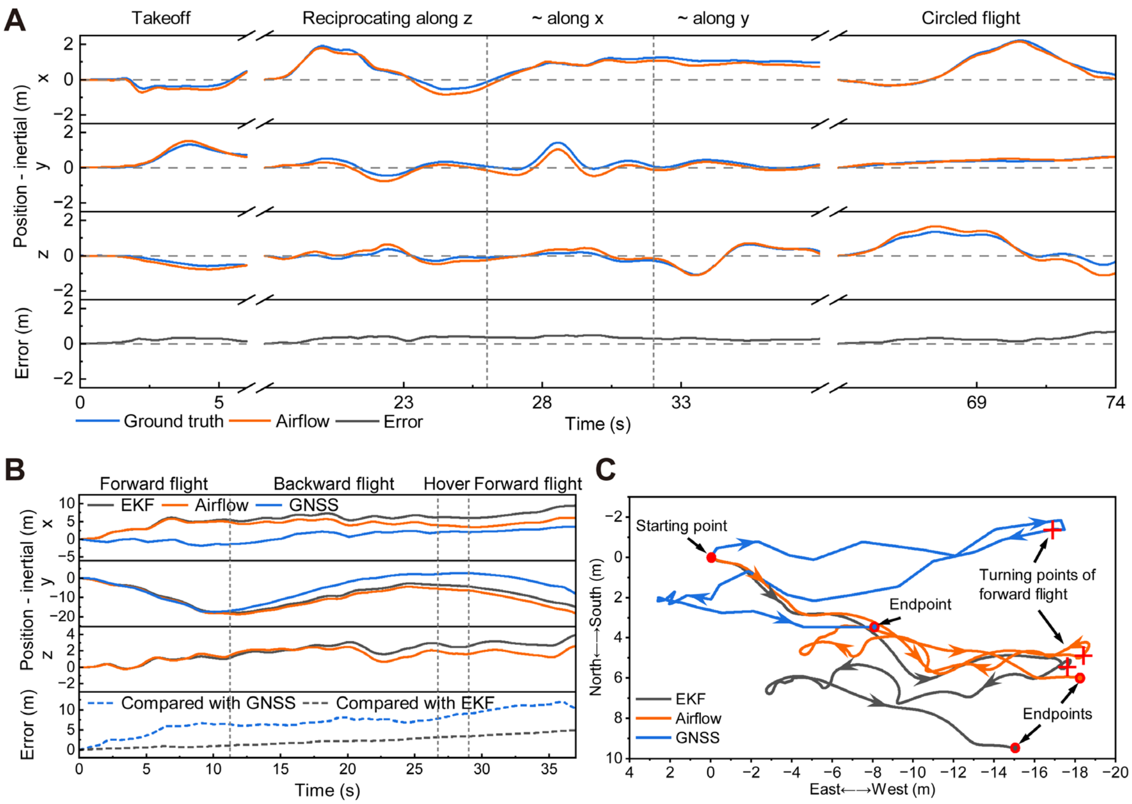

Supplementary Fig. 9. Flight path estimation and drift assessment. (A) Drift of path estimation obtained by flight velocity (Airflow) with respect to the ground truth given by motion capture during indoor flight phases. **(B)** Drift of the path estimation obtained by velocity integration with respect to the path based on EKF and GNSS during outdoor flight. **(C)** Path obtained by velocity integration versus the path given by EKF and GNSS during outdoor flight.

Supplementary Table 1 Comparison of the performance of existing flight parameter detection studies.

Sensor		Parameters estimation error						Ref.
Type	Number	*x*-velocity	*y*-velocity	*z*-velocity	Resultant-velocity	AOA	AOS	
Pressure	5	\	\	\	0.62 $m \cdot s^{-1}$	0.51°	\	[10]
Pressure	4	\	\	\	0.758 $m \cdot s^{-1}$ (RMS)	0.93° (RMS)	\	[12]
Pressure	24	\	\	\	0.4591 $m \cdot s^{-1}$	0.3399°	0.2549°	[15]
Velocity	3	\	\	\	0.27 $m \cdot s^{-1}$	0.87°	0.27°	[16]
Velocity	4	0.38 $m \cdot s^{-1}$ (RMS)	0.31 $m \cdot s^{-1}$ (RMS)	0.28 $m \cdot s^{-1}$ (RMS)	\	\	\	[17]
Velocity	2	0.097 $m \cdot s^{-1}$	0.141 $m \cdot s^{-1}$	0.145 $m \cdot s^{-1}$	0.151 $m \cdot s^{-1}$	0.58°	0.53°	Our

Supplementary Table 2 Thermal and electrical properties of common thermal sensor materials.

Material	TCR (% K^{-1})	Resistivity (Ω·m)	Ref.
Pm-Si_xGe_y:H	−6.6	2.8×10^5	[18]
$Ge_xSi_{1-x}O_y$	−5	3.8×10^2	[19]
YBCO	−3 ~ −4	3	[20]
a-Si:H	−2.8	~ 2	[21]
Germanium	−2	5	[22]
Copper-Graphene	0.313	2.03×10^{-8}	[23]
Platinum	0.39	1.06×10^{-8}	[24]
VO_x	−1.9	~ 0.3	Our

Supplementary Table 3 Comparison of the performance of existing calorimetric flow sensors.

Flexible	Sensing modality	Structure	Sensing element material	Maximum Sensitivity	Minimum detectable velocity	Repeatability error	Heating power	Max angle error	Detection range	Ref.
No	1D	Cavity	poly-Si / W-Ti	$0.017\ V\cdot m^{-1}\cdot s$	$0.8\ mm\cdot s^{-1}$	\	\	\	$0–20\ mm\cdot s^{-1}$	34
No	2D	Trench	Ni	$15.34\ mV\cdot m^{-1}\cdot s$	\	\	256 mW	5°	$0–33\ m\cdot s^{-1}$	36
No	2D	Solid	Pt	\	\	1.25%	\	2°	$0–40\ m\cdot s^{-1}$	37
No	2D	Suspended	poly-Si	$0.16\ V\cdot m^{-1}\cdot s$	\	4%	<10 mW	3°	$0–20\ m\cdot s^{-1}$	38
No	1D	Suspended	poly-Si	$0.59\ V\cdot m^{-1}\cdot s$	$2.5\ mm\cdot s^{-1}$	0.2%	<4.2 mW	\	$-23–23\ m\cdot s^{-1}$	39
No	1D	Suspended	poly-Si	$0.453\ V\cdot m^{-1}\cdot s$	$0.1\ mm\cdot s^{-1}$	\	<1.72 mW	\	$-6–6\ m\cdot s^{-1}$	40
Yes	2D	Solid	Pt	$1.2\ V^2\cdot m^{-0.5}\ s^{0.5}$	\	2%	160 mW	~2.1°	$3–30\ m\cdot s^{-1}$	41
Yes	2D	Suspended	VO_x	$1.817\ V\cdot m^{-1}\cdot s$	$0.11\ mm\cdot s^{-1}$	0.5%	< 4.4 mW	1.6°	$0–30\ m\cdot s^{-1}$	Our

Supplementary Table 4 Physical parameters required for calculations and CFD simulations.

Parameter	Symbol	Value
Overheated temperature of heater	T_h	20 K
Kinematic Viscosity	v	14.8×10^{-6} $m^2 \cdot s^{-1}$
Dynamic viscosity	μ	17.9×10^{-6} Pa·s
Thermal diffusion coefficient	a	22×10^{-6} $m^2 \cdot s^{-1}$
Fluid thermal conductivity	k_f	0.0267 $W \cdot (m \cdot k)^{-1}$
Fluid density	ρ	1.205 $Kg \cdot m^{-3}$
Fluid specific heat capacity	C_p	1004 $J \cdot (kg \cdot K)^{-1}$
Solid thermal conductivity (PI)	\	0.12 $W \cdot (m \cdot k)^{-1}$
Solid density (PI)	\	1420 $Kg \cdot m^{-3}$
Solid specific heat capacity (PI)	\	1090 $J \cdot (kg \cdot K)^{-1}$

Supplementary Table 5 Structure and training parameters of MLP neural networks.

Objects	Input layer nodes	Hidden layers nodes	Output layer nodes	Activation function	Learning rate	Optimization algorithm
AOA and AOS	PV1 (P1, V1)	3×50	AOA, AOS	ReLU	0.001	Adam
	PV2 (P2, V2)	3×50	AOA, AOS	ReLU	0.001	Adam
	PV3 (P3, V3)	3×50	AOA, AOS	ReLU	0.001	Adam
	PV1-3 (P1, P2, P3, V1, V2, V3)	3×50	AOA, AOS	ReLU	0.001	Adam
Relative airflow velocity	P1-2 and V1-2 of two FCF sensors (P1, P2, V1, V2, P1’, P2’, V1’, V2’)	3×100	Vx, Vy, Vz	ReLU	0.001	Adam

Supplementary Note 1 Derivation of heat transfer model for calorimetric sensors with suspended structure.

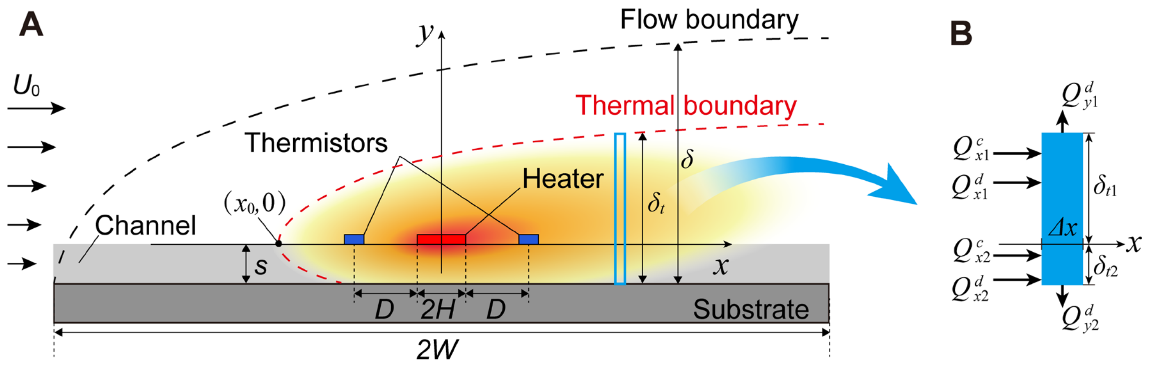

Supplementary Fig. 10. One-dimensional heat transfer model for calorimetric flow sensor with suspended structure. (A) Schematic of a general 1D model with the input velocity U_0. **(B)** Control volume of $\Delta x \times y$ (excluding the micro heater region) for heat transfer analysis.

According to the boundary layer theory[28], assuming that the free stream flow over the plane surface with velocity U_0 (Supplementary Fig. 10A), the flow velocity within the boundary layer can be expressed as:

$$U = U_0\left[\frac{3}{2}\frac{y+s}{\delta} - \frac{1}{2}\left(\frac{y+s}{\delta}\right)^3\right]; -s \le y \le \delta \tag{1}$$

Where s is the suspended height and δ is the fluid boundary layer height. The origin of the coordinate system is established at the center of the heater. We assume that the ambient temperature is T_0, the heating temperature is $T_0 + T_h$, the fluid temperature at (x, y) within the thermal boundary layer is $T(x, y) + T_0$, and thus the temperature on the x-axis is $T(x) + T_0$. Assuming that the thermal boundary layer δ_t is thinner than the momentum boundary layer δ, and the temperature within the thermal boundary layer can be fitted to a cubic polynomial, as in the case of the velocity profile[29]:

$$\frac{\left(T(x,y)+T_0\right)-\left(T(x)+T_0\right)}{T_0-\left(T(x)+T_0\right)} = \frac{3}{2}\frac{y}{\delta_t} - \frac{1}{2}\left(\frac{y}{\delta_t}\right)^3 \tag{2}$$

As shown in Supplementary Fig. 10B, we divide the thermal boundary layer into δ_{t1} and δ_{t2} according to the direction of the y-axis. From equation (2), we obtain the corresponding temperature as:

$$\begin{cases} T_1(x,y) = T(x)\left[1 - \frac{3}{2}\frac{y}{\delta_{t1}} + \frac{1}{2}\left(\frac{y}{\delta_{t1}}\right)^3\right]; 0 \le y \le \delta_{t1} \\ T_2(x,y) = T(x)\left[1 + \frac{3}{2}\frac{y}{\delta_{t2}} - \frac{1}{2}\left(\frac{y}{\delta_{t2}}\right)^3\right]; -\delta_{t2} \le y < 0 \end{cases} \tag{3}$$

Thus, we need to build the model to solve for the temperature $T(x)$ in the x-axis. In the laminar flow state, we assume that the temperature distribution of the fluid in the y-axis direction conforms to the boundary layer theory, ignore the temperature distribution perpendicular to the x-y plane, and consider only the overheated temperature $T(x)$ along the flow direction. Thus, the 3D heat transfer analysis is reduced to a simple 1D model. Considering the elemental control volume $\Delta x \times y$ shown in Supplementary Fig. 10B, the energy balance may be written as[30]:

$$Q_{x1}^d + Q_{x1}^v + Q_{x2}^d + Q_{x2}^v = Q_{y1}^d + Q_{y2}^d \tag{5}$$

Where the subscripts 1 and 2 denote the fluid above and below the x-axis, respectively, the superscripts d and v denote heat conduction and heat convection, respectively. The upper and lower bounds of the control volume depend on the thermal boundary layers δ_{t1} and δ_{t2}. The left side of equation (5) is the net heat input, which can be calculated as:

$$\begin{aligned} Q_{x1}^d &= -k_f \int_0^{\delta_{t1}} \frac{d}{dx}(T_1(x,y)+T_0)dy + k_f \int_0^{\delta_{t1}} \frac{d}{dx}\left(T_1(x,y)+T_0+\frac{d}{dx}(T_1(x,y)+T_0)\right)\Delta x dy \\ &= k_f \int_0^{\delta_{t1}} \frac{d^2}{dx^2}(T_1(x,y)+T_0)\Delta x dy \\ &= k_f \int_0^{\delta_{t1}} \frac{d^2}{dx^2}T(x)\left[1 - \frac{3}{2}\frac{y}{\delta_{t1}} + \frac{1}{2}\left(\frac{y}{\delta_{t1}}\right)^3\right]\Delta x dy \end{aligned} \tag{6-1}$$

$$
\begin{aligned}
Q_{x2}^{d} &= -k_f \int_{-\delta_{t2}}^{0} \frac{d}{dx}(T_2(x,y)+T_0)dy + k_f \int_{-\delta_{t2}}^{0} \frac{d}{dx}\left(T_2(x,y)+T_0+\frac{d}{dx}(T_2(x,y)+T_0) \right)\Delta x dy \\
&= k_f \int_{-\delta_{t2}}^{0} \frac{d^2}{dx^2}(T_2(x,y)+T_0)\Delta x dy \\
&= k_f \int_{-\delta_{t2}}^{0} \frac{d^2}{dx^2}T(x)\left[1+\frac{3}{2}\frac{y}{\delta_{t2}}-\frac{1}{2}\left(\frac{y}{\delta_{t2}}\right)^3\right]\Delta x dy
\end{aligned}
\tag{6-2}
$$

$$
\begin{aligned}
Q_{x1}^{v} &= \rho C_p \int_{0}^{\delta_{t1}} U(T_1(x,y)+T_0)dy - \rho C_p \int_{0}^{\delta_{t1}} U\left(T_1(x,y)+T_0+\frac{d}{dx}(T_1(x,y)+T_0) \right)\Delta x dy \\
&= -\rho C_p \int_{0}^{\delta_{t1}} U\frac{d}{dx}(T_1(x,y)+T_0)\Delta x dy \\
&= -\rho C_p \int_{0}^{\delta_{t1}} \left\{ U_0\left[\frac{3}{2}\frac{y+s}{\delta}-\frac{1}{2}\left(\frac{y+s}{\delta}\right)^3\right]\frac{d}{dx}T(x)\left[1-\frac{3}{2}\frac{y}{\delta_{t1}}+\frac{1}{2}\left(\frac{y}{\delta_{t1}}\right)^3\right]\right\}\Delta x dy
\end{aligned}
\tag{6-3}
$$

$$
\begin{aligned}
Q_{x2}^{v} &= \rho C_p \int_{-\delta_{t2}}^{0} U(T_2(x,y)+T_0)dy - \rho C_p \int_{-\delta_{t2}}^{0} U\left(T_2(x,y)+T_0+\frac{d}{dx}(T_2(x,y)+T_0) \right)\Delta x dy \\
&= -\rho C_p \int_{-\delta_{t2}}^{0} U\frac{d}{dx}(T_2(x,y)+T_0)\Delta x dy \\
&= -\rho C_p \int_{-\delta_{t2}}^{0} \left\{ U_0\left[\frac{3}{2}\frac{y+s}{\delta}-\frac{1}{2}\left(\frac{y+s}{\delta}\right)^3\right]\frac{d}{dx}T(x)\left[1+\frac{3}{2}\frac{y}{\delta_{t2}}-\frac{1}{2}\left(\frac{y}{\delta_{t2}}\right)^3\right]\right\}\Delta x dy
\end{aligned}
\tag{6-4}
$$

Where k_f denotes the fluid thermal conductivity, ρ is the fluid density, and C_p is the constant pressure specific heat capacity. The right side of equation (5) is the net heat output, which can be calculated as:

$$
Q_{y1}^{d} = -k_f \Delta x \left.\frac{\partial T_1(x,y)}{\partial y}\right|_0 \tag{7-1}
$$

$$
Q_{y2}^{d} = k_c k_f \Delta x \left.\frac{\partial T_2(x,y)}{\partial y}\right|_0 \tag{7-2}
$$

A compensation factor $k_c > 1$ is added to the Q_{y2}^{d} term to correct for the enhanced heat transfer capacity of the substrate as compared to air at $y = \delta_{t2}$[31]. After omitting the higher order quantities, they can be simplified as:

$$Q_{x1}^{d}=\frac{3}{8}k_f\left(\delta_{t1}\frac{d^2T(x)}{dx^2}+2\frac{dT(x)}{dx}\frac{d\delta_{t1}}{dx}+T(x)\frac{d^2\delta_{t1}}{dx^2}\right)\Delta x \tag{8-1}$$

$$Q_{x2}^{d}=\frac{3}{8}k_f\left(\delta_{t2}\frac{d^2T(x)}{dx^2}+2\frac{dT(x)}{dx}\frac{d\delta_{t2}}{dx}+T(x)\frac{d^2\delta_{t2}}{dx^2}\right)\Delta x \tag{8-2}$$

$$\begin{aligned}Q_{x1}^{v}=\frac{-\rho C_pU_0}{560\delta^3}\Bigg\{&\delta^2\left[(315s\delta_{t1}+84\delta_{t1}^2)\frac{dT(x)}{dx}+(315s+168\delta_{t1})\frac{d\delta_{t1}}{dx}T(x)\right]\\&-\Bigg[(6\delta_{t1}^4+84s^2\delta_{t1}^2+35s\delta_{t1}^3+105s^3\delta_{t1})\frac{dT(x)}{dx}\\&+(24\delta_{t1}^3+105s^3+105s\delta_{t1}^2+168s^2\delta_{t1})\frac{d\delta_{t1}}{dx}T(x)\Bigg]\Bigg\}\Delta x\end{aligned} \tag{8-3}$$

$$\begin{aligned}Q_{x2}^{v}=\frac{-\rho C_pU_0}{560\delta^3}\Bigg\{&\delta^2\left[(315s\delta_{t2}-84\delta_{t2}^2)\frac{dT(x)}{dx}+(315s-168\delta_{t2})\frac{d\delta_{t2}}{dx}T(x)\right]\\&+\Bigg[(6\delta_{t2}^4+84s^2\delta_{t2}^2-35s\delta_{t2}^3-105s^3\delta_{t2})\frac{dT(x)}{dx}\\&+(24\delta_{t2}^3-105s^3-105s\delta_{t2}^2+168s^2\delta_{t2})\frac{d\delta_{t2}}{dx}T(x)\Bigg]\Bigg\}\Delta x\end{aligned} \tag{8-4}$$

$$Q_{y1}^{d}=k_f\Delta x\frac{3T(x)}{2\delta_{t1}} \tag{8-5}$$

$$Q_{y2}^{d}=k_ck_f\Delta x\frac{3T(x)}{2\delta_{t2}} \tag{8-6}$$

By substituting equations (8-1) ~ (8-6) into equation (5), the linearized 1D model of the suspended structural calorimetric flow sensor can be obtained[30]:

$$A\frac{d^2T(x)}{dx^2}+B\frac{dT(x)}{dx}+CT(x)=0 \tag{9}$$

Where *A*, *B*, and *C* are written as:

$$A=\frac{3}{8}k_f\left(\delta_{t1}+\delta_{t2}\right) \tag{10-1}$$

$$B = \frac{3}{4}k_f\left(\frac{d\delta_{t1}}{dx} + \frac{d\delta_{t2}}{dx}\right) - \frac{\rho C_p U_0}{560\delta^3}\Big[\delta^2(315s\delta_{t1} + 84\delta_{t1}^2 + 315s\delta_{t2} - 84\delta_{t2}^2) - (6\delta_{t1}^4 + 84s^2\delta_{t1}^2 + 35s\delta_{t1}^3 + 105s^3\delta_{t1}) + (6\delta_{t2}^4 + 84s^2\delta_{t2}^2 - 35s\delta_{t2}^3 - 105s^3\delta_{t2})\Big] \tag{10-2}$$

$$C = \frac{3}{8}k_f\left(\frac{d^2\delta_{t1}}{dx^2} + \frac{d^2\delta_{t2}}{dx^2}\right) - \frac{\rho C_p U_0}{560\delta^3}\Big[\delta^2(168\delta_{t1} + 315s)\frac{d\delta_{t1}}{dx} + \delta^2(315s - 168\delta_{t2})\frac{d\delta_{t2}}{dx} + (24\delta_{t1}^3 + 105s^3 + 105s\delta_{t1}^2 + 168s^2\delta_{t1})\frac{d\delta_{t1}}{dx} + (24\delta_{t2}^3 - 105s^3 - 105s\delta_{t2}^2 + 168s^2\delta_{t2})\frac{d\delta_{t2}}{dx}\Big] - k_f\frac{3}{2\delta_{t1}} - k_c [illegible] \tag{10-3}$$

For the open-field perception model, the momentum boundary layer δ develops from the leading edge of the substrate ($-W$) and can be written as[28]:

$$\delta = \left[\frac{280}{13}\frac{v(x+W)}{U_0}\right]^{1/2} \tag{11}$$

Where v is the kinematic viscosity of the fluid. The heater is suspended at the center of the substrate and the thermal boundary layer δ_t starts from $x = x_0$[28]. Furthermore, the suspended height s is small relative to the thermal boundary layer, and the thermal boundary layer δ_{t2} in the lower part of the heater can be simplified to s; the thermal boundary layer in the upper part of the heater δ_{t1} can be determined from δ. Thus, the thermal boundary layer can be written as:

$$\delta_{t1} = \frac{\delta}{1.026}\left(\frac{v}{a}\right)^{-1/3}\left[1 - \left(\frac{x_0 + W}{x + W}\right)^{3/4}\right]^{1/3} + s; x \geq x_0 \tag{12-1}$$

$$\delta_{t2} = s \tag{12-2}$$

Where a is the thermal diffusion coefficient of the fluid. Due to the simplification of the thermal boundary layer δ_{t2}, A, B, and C in equation (10) can be simplified as:

$$A = \frac{3}{8}k_f\left(\delta_{t1} + s\right) \tag{13-1}$$

$$B = \frac{3}{4}k_f\frac{d\delta_{t1}}{dx} - \frac{\rho C_p U_0}{560\delta^3}\Big[\delta^2(315s\delta_{t1} + 84\delta_{t1}^2 + 231s^2) - (6\delta_{t1}^4 + 84s^2\delta_{t1}^2 + 35s\delta_{t1}^3 + 105s^3\delta_{t1}) - 50s^4\Big] \tag{13-2}$$

$$C=\frac{3}{8}k_f\frac{d^2\delta_{t1}}{dx^2}-\frac{\rho C_p U_0}{560\delta^3}\left[\delta^2(168\delta_{t1}+315s)\frac{d\delta_{t1}}{dx}\right.$$
$$\left.+(24\delta_{t1}^3+105s^3+105s\delta_{t1}^2+168s^2\delta_{t1})\frac{d\delta_{t1}}{dx}\right]-k_f\frac{3}{2\delta_{t1}}-k_c k_f\frac{3}{2s} \tag{13-3}$$

The calculation of the temperature distribution starts at the starting point of the thermal boundary layer $x = x_0$. To simplify the calculation, we assume that the fluid temperature at the symmetric position $x = -x_0$ is equal to the ambient temperature T_0. The temperature at the heater is $T_h + T_0$. Therefore, the boundary condition for the overheated temperature $T(x)$ is satisfied as:

$$T(x_0)=T(-x_0)=0;T(-H)=T(H)=T_h \tag{14}$$

Applying the thermal boundary conditions of equation (14) to equations (9) and (13), we can establish the analytical solution of overheated temperature $T(x)$ on the x-axis of the flow direction as follows:

$$\begin{cases} T(x)=\dfrac{T_h(e^{r_2x}-e^{r_2x_0+r_1(x-x_0)})}{e^{-r_2H}-e^{r_2x_0-r_1(H+x_0)}};x_0\le x<-H \\ T(x)=T_h;-H\le x\le H \\ T(x)=\dfrac{T_h(e^{r_2x}-e^{-r_2x_0+r_1(x+x_0)})}{e^{r_2H}-e^{-r_2x_0+r_1(H+x_0)}};H<x\le -x_0 \end{cases} \tag{15-1}$$

$$r_{1,2}=\frac{-B\pm\sqrt{B^2-4AC}}{2A} \tag{15-2}$$

Assuming two thermistor sensors at $x = D + H$ and $-x = -D - H$, the thermal output $\Delta T(x)$ of FCF sensor can be determined as:

$$\Delta T(x)=T\left(x\right)-T\left(-x\right) \tag{16}$$

In the calculation, W is set to 5 cm, which is half the width of our FCF sensor. H is set to 0.1 mm, and x_0 is set to −2.5 mm, which is the distance between the Cu channel and the center heater of the FCF sensor. The compensation factor k_c is 1.3, which corresponds well with the simulation results. The other physical parameters required to calculate $T(x)$ are shown in Supplementary Table 4.
