# Supplementary material for: Flexible calorimetric flow sensor with unprecedented sensitivity and directional resolution for multiple flight parameter detection
Source: Nat Commun. 2024 Apr 10;15:3091. doi: 10.1038/s41467-024-47284-7 (PMC11006672; doi:10.1038/s41467-024-47284-7)
Supplement: Supplementary file 3 — Description of Additional Supplementary Files [file 41467_2024_47284_MOESM3_ESM.pdf]

DESCRIPTION OF ADDITIONAL SUPPLEMENTARY FILES

Supplementary Movie 1 : MAV indoor flight.

Supplementary Movie 2 : MAV outdoor flight.
