# Supplementary material for: Flexible calorimetric flow sensor with unprecedented sensitivity and directional resolution for multiple flight parameter detection
Source: Nat Commun. 2024 Apr 10;15:3091. doi: 10.1038/s41467-024-47284-7 (PMC11006672; doi:10.1038/s41467-024-47284-7)
Supplement: Supplementary file 6 — Reporting Summary [file 41467_2024_47284_MOESM6_ESM.pdf]

nature portfolio

Corresponding author(s): Yonggang Jiang

Last updated by author(s): Feb 20, 2024

Reporting Summary

Nature Portfolio wishes to improve the reproducibility of the work that we publish. This form provides structure for consistency and transparency in reporting. For further information on Nature Portfolio policies, see our Editorial Policies and the Editorial Policy Checklist.

Statistics

For all statistical analyses, confirm that the following items are present in the figure legend, table legend, main text, or Methods section.

n/a	Confirmed	
☐	☒	The exact sample size (*n*) for each experimental group/condition, given as a discrete number and unit of measurement
☐	☒	A statement on whether measurements were taken from distinct samples or whether the same sample was measured repeatedly
☒	☐	The statistical test(s) used AND whether they are one- or two-sided<br>*Only common tests should be described solely by name; describe more complex techniques in the Methods section.*
☒	☐	A description of all covariates tested
☒	☐	A description of any assumptions or corrections, such as tests of normality and adjustment for multiple comparisons
☐	☒	A full description of the statistical parameters including central tendency (e.g. means) or other basic estimates (e.g. regression coefficient) AND variation (e.g. standard deviation) or associated estimates of uncertainty (e.g. confidence intervals)
☒	☐	For null hypothesis testing, the test statistic (e.g. *F*, *t*, *r*) with confidence intervals, effect sizes, degrees of freedom and *P* value noted<br>*Give P values as exact values whenever suitable.*
☒	☐	For Bayesian analysis, information on the choice of priors and Markov chain Monte Carlo settings
☒	☐	For hierarchical and complex designs, identification of the appropriate level for tests and full reporting of outcomes
☒	☐	Estimates of effect sizes (e.g. Cohen's *d*, Pearson's *r*), indicating how they were calculated

Our web collection on statistics for biologists contains articles on many of the points above.

Software and code

Policy information about availability of computer code

Data collection	Labview 2018 was used to collect experimental data.
Data analysis	MATLAB R2020a was used to analyze heat transfer models.<br>ANSYS2020-R2 was used to CFD simulation analysis.<br>OriginLab was used to make plots.<br>The codes for training and testing MLP network models have been deposited in Github repository: https://github.com/by1907047/MLP.

For manuscripts utilizing custom algorithms or software that are central to the research but not yet described in published literature, software must be made available to editors and reviewers. We strongly encourage code deposition in a community repository (e.g. GitHub). See the Nature Portfolio guidelines for submitting code & software for further information.

Data

Policy information about availability of data

All manuscripts must include a data availability statement. This statement should provide the following information, where applicable:

- Accession codes, unique identifiers, or web links for publicly available datasets
- A description of any restrictions on data availability
- For clinical datasets or third party data, please ensure that the statement adheres to our policy

The datasets for training and testing MLP network models are available in figshare with the identifier doi: 10.6084/m9.figshare.24925971

Research involving human participants, their data, or biological material

Policy information about studies with human participants or human data. See also policy information about sex, gender (identity/presentation), and sexual orientation and race, ethnicity and racism.

Reporting on sex and gender	No data related to sex or gender was involved in this study.
Reporting on race, ethnicity, or other socially relevant groupings	No data related to race, ethnicity, or other socially relevant groupings was involved in this study.
Population characteristics	No data related to population characteristics was involved in this study.
Recruitment	No data related to recruitment was involved in this study.
Ethics oversight	No data related to ethics was involved in this study.

Note that full information on the approval of the study protocol must also be provided in the manuscript.

Field-specific reporting

Please select the one below that is the best fit for your research. If you are not sure, read the appropriate sections before making your selection.

☐ Life sciences ☐ Behavioural & social sciences ☒ Ecological, evolutionary & environmental sciences

For a reference copy of the document with all sections, see nature.com/documents/nr-reporting-summary-flat.pdf

Ecological, evolutionary & environmental sciences study design

All studies must disclose on these points even when the disclosure is negative.

Study description	FCF sensor characteristics and flight parameter detection capabilities are investigated in this paper.
Research sample	We tested the characteristics of the FCF sensor including output at different flow velocities, repeatability, response time, and directionality. We tested flight parameter detection including AOA, AOS, flight velocity, and wing vibration.
Sampling strategy	There is no such problem about sampling in this study.
Data collection	All data characterizing the sensors were collected via the data acquisition card NI-6366. All flight experiment data was collected via the on-board microcontroller ESP32-2S.
Timing and spatial scale	The data within each dataset is collected continuously with a sampling frequency of 50 Hz.
Data exclusions	No data were excluded from the analyses.
Reproducibility	For the sensor characterization tests, we tested 3 times under the same conditions and calculated the mean and standard deviation of the test results for each condition.
Randomization	In order to assess the performance of estimation over a larger angular range and typical flight paths, the testing sets for angular estimation and flight experiments were selected artificially rather than randomly.
Blinding	Data from the test set used to test the performance of the model is not included in the training set for training the model.

Did the study involve field work? ☐ Yes ☒ No

Reporting for specific materials, systems and methods

We require information from authors about some types of materials, experimental systems and methods used in many studies. Here, indicate whether each material, system or method listed is relevant to your study. If you are not sure if a list item applies to your research, read the appropriate section before selecting a response.

Materials & experimental systems

n/a	Involved in the study
☒	☐ Antibodies
☒	☐ Eukaryotic cell lines
☒	☐ Palaeontology and archaeology
☒	☐ Animals and other organisms
☒	☐ Clinical data
☒	☐ Dual use research of concern
☒	☐ Plants

Methods

n/a	Involved in the study
☒	☐ ChIP-seq
☒	☐ Flow cytometry
☒	☐ MRI-based neuroimaging

Plants

Seed stocks	This study does not involve plants.
Novel plant genotypes	This study does not involve plants.
Authentication	This study does not involve plants.
